# Encephalitis Hospitalization Rates and Inpatient Mortality in the United States, 2000-2010

**DOI:** 10.1371/journal.pone.0104169

**Published:** 2014-09-05

**Authors:** Benjamin P. George, Eric B. Schneider, Arun Venkatesan

**Affiliations:** 1 Center for Surgical Trials and Outcomes Research, Department of Surgery, Johns Hopkins School of Medicine, Baltimore, Maryland, United States of America; 2 University of Rochester School of Medicine and Dentistry, Rochester, New York, United States of America; 3 Johns Hopkins Encephalitis Center, Department of Neurology, Johns Hopkins School of Medicine, Baltimore, Maryland, United States of America; California Department of Public Health, United States of America

## Abstract

**Background:**

Encephalitis rates by etiology and acute-phase outcomes for encephalitis in the 21^st^ century are largely unknown. We sought to evaluate cause-specific rates of encephalitis hospitalizations and predictors of inpatient mortality in the United States.

**Methods:**

Using the Nationwide Inpatient Sample (NIS) from 2000 to 2010, a retrospective observational study of 238,567 patients (mean [SD] age, 44.8 [24.0] years) hospitalized within non-federal, acute care hospitals in the U.S. with a diagnosis of encephalitis was conducted. Hospitalization rates were calculated using population-level estimates of disease from the NIS and population estimates from the *United States Census Bureau*. Adjusted odds of mortality were calculated for patients included in the study.

**Results:**

In the U.S. from 2000–2010, there were 7.3±0.2 encephalitis hospitalizations per 100,000 population (95% CI: 7.1–7.6). Encephalitis hospitalization rates were highest among females (7.6±0.2 per 100,000) and those <1 year and >65 years of age with rates of 13.5±0.9 and 14.1±0.4 per 100,000, respectively. Etiology was unknown for approximately 50% of cases. Among patients with identified etiology, viral causes were most common (48.2%), followed by Other Specified causes (32.5%), which included predominantly autoimmune conditions. The most common infectious agents were herpes simplex virus, toxoplasma, and West Nile virus. Comorbid HIV infection was present in 7.7% of hospitalizations. Average length of stay was 11.2 days with mortality of 5.6%. In regression analysis, patients with comorbid HIV/AIDS or cancer had increased odds of mortality (odds ratio [OR]  = 1.70; 95% CI: 1.30–2.22 and OR = 2.26; 95% CI: 1.88–2.71, respectively). Enteroviral, postinfectious, toxic, and Other Specified causes were associated with lower odds vs. herpes simplex encephalitis.

**Conclusions:**

While encephalitis and encephalitis-related mortality impose a considerable burden in the U.S. in the 21^st^ Century, the reported demographics of hospitalized encephalitis patients may be changing.

## Introduction

Acute encephalitis is a challenging syndrome to diagnose and manage given the heterogeneity of clinical presentations and the myriad of causative agents. Over the past decade, numerous advances have uncovered novel infectious and autoimmune etiologies of encephalitis. Despite such advances, in large studies more than 50% of encephalitis cases typically remain without an identified etiology, posing additional challenges in delivering prognosis and treatment [1]–[4].

In the United States, encephalitis has been associated with a substantial disease burden and considerable mortality [4]–[6]. In the last decade, West Nile Virus has emerged as an important viral cause of encephalitis [7], [8], the demographics of the HIV/AIDS epidemic have changed dramatically [9], [10], and there is increasing focus by clinicians and researchers on non-infectious etiologies [11]–[16]. Additionally, the general U.S. healthcare environment has evolved and there is increasing scrutiny of patient outcomes [17], [18]. Here, we sought to investigate population-based estimates of encephalitis in the United States between 2000 and 2010 to elucidate the contribution of specific etiologies to the current landscape of encephalitis. In addition, we sought to examine acute-phase outcomes of U.S. encephalitis cases including inpatient death and predictors of mortality.

## Methods

### Ethics

This study employed anonymized limited datasets provided by the United States Agency for Healthcare Research and Quality (AHRQ) Healthcare Costs and Utilization Project (HCUP) under the terms of a formal data use agreement [19]. This research was approved by the Institutional Review Board of the Johns Hopkins Medical Institutions.

### Study Sample

A retrospective study of nationally representative hospital discharges for patients admitted to acute inpatient care for encephalitis between 2000–2010 was performed using the Agency for Healthcare Research and Quality (AHRQ) Healthcare Costs and Utilization Project (HCUP) Nationwide Inpatient Sample (NIS) dataset [19]. The NIS provides a 20% sample of all inpatient hospital discharges and can be weighted to represent the entire U.S. population, which was done for this study. The NIS dataset is a cross-sectional, all-payer, inpatient care dataset in the U.S., consolidated on an annual basis, containing information on approximately 8 million hospitalizations annually from 1,051 hospitals in 45 states. It is the only national hospital database that includes persons covered by Medicare, Medicaid, private insurance, as well as uninsured patients. The sampling frame embodied 95% of all U.S. hospital discharges in 2010. Discharge data provided in the NIS includes patient demographic information as well as information on diagnosis, geographic location, payer status, hospital charges, length of stay, discharge disposition, and in-hospital death. Information on hospital characteristics, such as teaching status and bed size, can be matched to individual hospitalizations; however, individual patient identifiers and patient death records are not available.

### Encephalitis and Accompanying Diagnoses

Patients admitted to inpatient care with encephalitis were identified using *International Classification of Disease*, Ninth Revision (ICD-9) diagnosis codes and categorized by etiology (**Table 1**). Patient demographic, hospital and regional variables were obtained from NIS data. The presence of comorbidities and complications were determined using ICD-9 codes. A Charlson comorbidity index score was calculated for each patient to account for a broad range of comorbid illness [20], [21]. The Charlson comorbidity index is a weighted index based on the presence of 17 accompanying diagnoses or comorbid diseases. Each comorbid disease is assigned a weight from 1 to 6; the index is the sum of the weights and can range from 0 to 33. For the purposes of this study, mild was assigned a score of 0, moderate a score of 1, and severe a score greater than or equal to 2.

**Table 1:** Diagnosis codes for encephalitis.

Encephalitis Diagnosis	ICD-9 Codes
*Herpetic meningoencephalitis (HSV-1/HSV-2)*	054.3
*Enteroviral encephalitis^a^*	048
*Postvaricella encephalitis (VZV)*	052.0
*Other human herpes encephalitis (Other HHV)*	058.2
*Other viral*	
Acute bulbar poliomyelitis	045.0
Subacute sclerosing panencephalitis	046.2
Other specified non-arthropodborne encephalitides	049.8
Encephalomyelitis due to rubella	056.01
Australian encephalitis	062.4
Russian spring-summer encephalitides	063.0
Louping ill	063.1
Central European encephalitis	063.2
Other specified tickborne viral encephalitis	063.8
Rabies	071
Mumps encephalitis	072.2
Encephalitis in viral diseases classified elsewhere	323.0
*West Nile*	066.41
*Arboviral*	
Japanese encephalitis	062.0
Western equine encephalitis	062.1
Eastern equine encephalitis	062.2
St. Louis encephalitis	062.3
California virus encephalitis	062.5
Other specified mosquitoborne viral encephalitis	062.8
Venezuelan equine encephalitis	066.2
*Postinfectious*	
Postmeasles encephalitis	055.0
Postinfectious encephalitis^b^	323.6
*Toxoplasmic*	130.0
*Meningococcal*	036.1
*Other infectious*	
Tuberculous encephalitis	013.6
Congenital syphilitic encephalitis	090.41
Syphilitic encephalitis	094.81
Meningoencephalitis due to Naegleria species	136.29
Encephalitis in rickettsial diseases classified elsewhere	323.1
Encephalitis in protozoal disease classified elsewhere	323.2
Other encephalitides due to infection classified elsewhere	323.4
*Postimmunization*	
Encephalitis following immunization procedures	323.5
*Toxic*	323.7
*Other specified^c^*	323.8
*Unspecified*	
Unspecified cause of encephalitis	323.9
Unspecified mosquitoborne encephalitis	062.9
Unspecified tickborne encephalitis	063.9
*Viral encephalitis not otherwise specified*	
Viral encephalitis not otherwise specified	049.9
Viral encephalitis transmitted by other and unspecified arthropods	064

Abbreviations: HSV  =  Herpes Simplex Virus; VZV  =  Varicella Zoster Virus; HHV  =  Human Herpesvirus.

*a) Applies to enteroviruses diseases of central nervous system other than meningitis (ICD-9 047).*

b) Postinfectious encephalitis applies to infectious acute disseminated encephalomyelitis (ADEM).

c) Other specified encephalitis applies to noninfectious acute disseminated encephalomyelitis (ADEM), allergic encephalitis, autoimmune encephalitis, Bickerstaff's brainstem encephalitis, eosinophilic meningoencephalitis, paraneoplastic limbic encephalitis, and systemic lupus erythematosus encephalitis.

### Missing Data

In general, variables with less than 1% missing data from the sample were evaluated after the exclusion of the missing data points. Missing data that were excluded included that for sex (n = 113), primary payer (n = 75), and hospital location/teaching status (n = 200). Race had 23% missing data due to suppression of the variable by certain states and hospitals within the dataset. Therefore, race was not included as a variable within the primary analysis to avoid the need for a large exclusion or imputation.

### Outcomes and Covariates

To evaluate burden of disease, we calculated U.S. hospitalization rates using data from the NIS and the *United States Census Bureau* (2000–2010) and examined annual trends from 2000–2010. Population-based encephalitis admission rates were studied across sex, age group, and region, both overall and by etiology. Hospitalization rates were additionally evaluated across five-year age groups for a more detailed examination of age distribution by encephalitis etiology.

Encephalitis hospitalizations over the 11-year period were evaluated by admission month in order to examine seasonal distribution of viral and viral “not otherwise specified” etiologies. Data on admission month were missing for 8.7% of encephalitis admissions examined and were therefore excluded from the analysis on seasonality. The aggregated analysis summing all seasonal data from 2000–2010 may be limited by the introduction of ICD-9 codes for West Nile Virus in 2004. Therefore, a sensitivity analysis was conducted using only data from 2005–2010.

The primary outcome in this study was in-hospital mortality. Mortality was examined in univariate analysis by various patient characteristics and encephalitis subgroups. Predictors of mortality were assessed using forced entry binary multivariable logistic regression modeling. Covariates included in the final model were sex, age, cancer, HIV/AIDS, Charlson comorbidity index, hospital region and location/teaching status, and etiology. Complications were excluded from the model examining mortality. Primary payer was excluded from the model due to an imbalanced association with age (88% of those ≥65 years were Medicare enrollees). Race was excluded due to missing data (23%). Unspecified, other viral, other infectious, viral NOS, and causes of encephalitis were excluded from the regression analysis due to heterogeneity or ambiguity of each group (see: **Table 1**). In addition, Toxoplasmic encephalitis was excluded due to 95% concordance with HIV/AIDS.

### Statistical Analysis

Descriptive statistics were used to assess encephalitis hospitalization rates by patient characteristics as well as to examine outcomes across several variables. To generate national estimates of encephalitis hospitalizations, frequencies obtained from the NIS were scaled using HCUP discharge weights. Standard errors of estimation were derived from NIS sampling techniques based on NIS strata and hospital sampling units. The χ^2^ test was used for categorical analysis where applicable. An adjusted Wald test was used to compare number of admissions across seasons and differences in mean values (e.g., length of stay).

For univariate and multivariate analyses, odds ratios and standard errors were calculated for all variables in the model. Significance was set a priori at *P*<0.05. All statistical tests and analyses were performed using the Stata version 12.0 (StataCorp, College Station, TX).

## Results

### Study Sample

Between January 1, 2000 and December 31, 2010, nearly one-quarter of a million (n = 238,706; 95% CI: 223,114–254,019) patients were admitted to acute inpatient hospital care in the United States with a diagnosis of encephalitis. Approximately 53% of encephalitis patients were female and the average age was 44.8 years (SD = 24.0). Among all encephalitis admissions, 7.7% were found to have comorbid HIV/AIDS while 6.8% had cancer (**Table 2**).

**Table 2:** Encephalitis cohort demographics, 2000–2010.

Patient Characteristics	Unweighted Hospitalizations (n = 48,596)	Population Estimated Hospitalizations ± SE (n = 238,567)		Percentage
Sex				
Male	22,898	112,315	±3,870	(47.2)
Female	25,585	125,694	±4,130	(52.8)
Age group, years				
<1	1,207	5,896	±455	(2.5)
1–4	1,814	8,878	±726	(3.7)
5–19	6,087	29,898	±1,987	(12.5)
20–44	13,688	67,117	±2,658	(28.1)
45–64	14,107	69,334	±2,332	(29.1)
≥65	11,693	57,444	±1,638	(24.1)
Race^a^				
White	23,000	112,968	±3,326	(47.4)
Black	7,114	34,792	±1,967	(14.6)
Hispanic	4,684	22,754	±1,417	(9.5)
Asian, Pacific Islander, Native American, or other	2,660	13,090	±689	(5.5)
Missing	11,138	54,963	±3,143	(23.0)
Primary payer*				
Medicare	14,698	72,173	±2,082	(30.3)
Private	19,906	97,896	±3,589	(41.1)
Medicaid	9,196	45,138	±2,435	(18.9)
Self pay, no charge, or other	4,721	22,997	±1,234	(9.7)
Charlson Comorbidity Index^b^				
Mild	22,937	112,496	±3,942	(47.2)
Moderate	10,156	49,826	±1,582	(20.9)
Severe	15,503	76,245	±3,052	(32.0)
Comorbidities^c^				
Acute Myocardial Infarction	1,159	5,718	±253	(2.4)
Congestive Heart Failure	2,375	11,652	±417	(4.9)
Cerebrovascular Disease	5,045	24,781	±890	(10.4)
COPD	4,906	24,169	±766	(10.1)
Rheumatoid Disease	4,606	22,573	±998	(9.5)
Diabetes^d^	6,375	29,772	±929	(12.5)
Renal Disease	2,483	12,266	±541	(5.1)
Non-metastatic Carcinoma	2,292	11,346	±576	(4.8)
Metastatic Carcinoma	972	4802	±246	(2.0)
HIV/AIDS	3,813	18,400	±1,680	(7.7)
Hospital Region				
Northeast	8,675	44,460	±3,470	(18.6)
Midwest	10,449	52,782	±3,107	(22.1)
South	19,218	91,957	±5,591	(38.5)
West	10,254	49,368	±3,025	(20.7)
Hospital Location/Teaching Status				
Rural	4,241	21,240	±1,281	(8.9)
Urban, Nonteaching	16,269	78,636	±2,845	(33.0)
Urban, Teaching	27,886	137,727	±7,749	(57.7)
Encephalitis Cause				
HSV-1/HSV-2	6,698	32,918	±1,087	(13.8)
Enteroviral	2,796	13,698	±587	(5.7)
VZV	192	950	±86	(0.4)
Other HHV	263	1,313	±105	(0.5)
Arboviral	209	1,019	±120	(0.4)
West Nile	713	3,513	±218	(1.5)
Other viral	1,555	7,612	±372	(3.2)
Toxoplasmic	1,416	6,934	±1,323	(2.9)
Meningococcal	316	1,554	±106	(0.7)
Other infectious	648	3,199	±175	(1.3)
Postinfectious	2,321	11,421	±707	(4.8)
Postimmunization	92	447	±51	(0.2)
Toxic	189	936	±83	(0.4)
Other specified	8,385	41,192	±1,698	(17.3)
Viral NOS	5,870	28,788	±937	(12.1)
Unspecified	16,962	83,212	±2,688	(34.9)

Abbreviations: SE  =  standard error; HIV/AIDS  =  Human Immunodeficiency Virus/Acquired Immunodeficiency Syndrome; HSV  =  Herpes Simplex Virus; VZV  =  Varicella Zoster Virus; HHV  =  Human Herpesvirus; NOS  =  not otherwise specified.

a) Variables with >1% missing values were included as “missing” within the table. Missing sex (n = 113), primary payer (n = 75), and hospital location/teaching status (n = 200) were not shown.

b) The Charlson Comorbidity Index is a weighted index based on the presence of 17 comorbid conditions. Each comorbid disease is assigned a weight from 1 to 6; the index is the sum of the weights for each comorbid condition and can range from 0 to 33. For the purposes of this study, mild was score  =  0, moderate score  =  1, and severe score ≥ 2.

c) Comorbidities affecting greater than 2% of encephalitis hospitalizations are displayed in the table. Others included within the index are hemiplegia/paraplegia, peripheral vascular disease, dementia, peptic ulcer disease, mild liver disease, and moderate to severe liver disease.

d) Diabetes was combined to include diabetes with and without complications; two separate conditions within the Charlson Comorbidity Index.

### Hospitalization for Encephalitis

**Figure 1:**
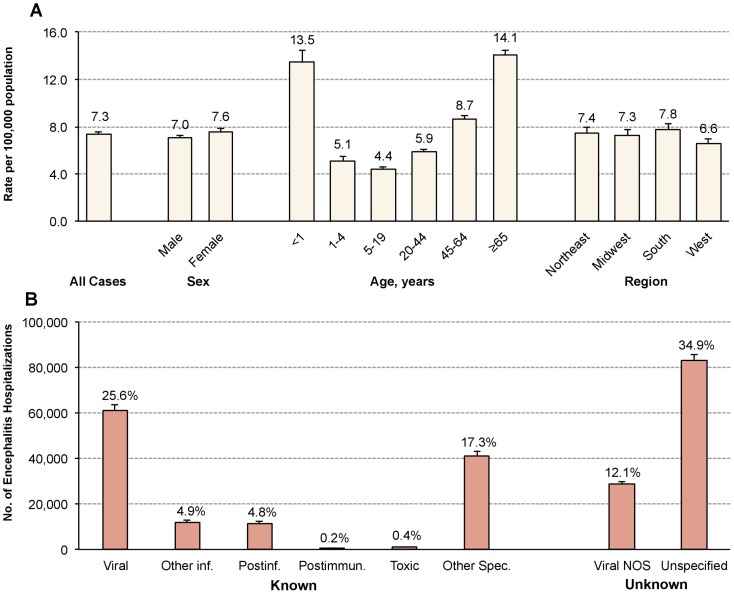
Encephalitis hospitalization rates^a^, 2000–2010: (A) by sex, age, and region-specific rates and (B) disease category. Abbreviations: NOS  =  not otherwise specified; inf.  =  infectious; Postimmun.  =  Postimmunization; Spec.  =  Specified. a) Population rates are calculated using hospitalization estimates from the Nationwide Inpatient Sample and population-specific estimates from the *United States Census Bureau*.

At the population level, the overall U.S. encephalitis hospitalization rate from 2000–2010 was 7.3 per 100,000 (95% CI: 7.1–7.6). Trends over the time period were also examined and can be found in **Table 3**. Overall encephalitis hospitalization rates increased from 6.6 per 100,000 (95% CI: 6.2–7.0) in 2000 to 7.6 per 100,000 (95% CI: 7.2–8.1) in 2010. Hospitalization rates were highest among females, older adults (≥65 years) and infants less than 1 year of age (**Figure 1A**). Across age groups, rates of encephalitis increased steadily from a low of 4.1 per 100,000 at 10–14 years of age and peaked at 15.7 per 100,000 at 75–79 years of age.

**Table 3:** Trends in encephalitis-associated hospitalization rates, 2000–2010.

	Hospitalization rate per 100,000 population											Annual Growth Rate
	2000	2001	2002	2003	2004	2005	2006	2007	2008	2009	2010	
**Cause**												
HSV-1/HSV-2	0.89	0.86	0.96	1.04	1.05	1.17	1.08	1.04	0.96	0.96	1.11	2.2%
Enteroviral	0.34	0.43	0.46	0.53	0.44	0.49	0.34	0.36	0.34	0.38	0.52	4.4%
VZV	0.05	0.04	0.04	0.03	0.03	0.03	0.02	0.02	0.01	0.03	0.03	−5.3%
Other HHV	NA	NA	NA	NA	NA	NA	NA	0.03	0.11	0.15	0.14	NA
Arboviral	0.01	0.01	0.10	0.03	0.02	0.04	0.03	0.02	0.02	0.02	0.04	20.1%
West Nile	NA	NA	NA	NA	0.04	0.25	0.30	0.19	0.15	0.10	0.14	NA
Other viral	0.23	0.21	0.26	0.25	0.22	0.25	0.23	0.20	0.25	0.21	0.26	1.3%
Toxoplasmic	0.33	0.33	0.26	0.29	0.25	0.14	0.13	0.20	0.20	0.10	0.14	−8.4%
Meningococcal	0.05	0.05	0.05	0.04	0.04	0.04	0.05	0.06	0.04	0.07	0.06	1.7%
Other infectious	0.07	0.07	0.09	0.10	0.06	0.07	0.10	0.11	0.12	0.16	0.13	5.9%
Postinfectious	0.26	0.29	0.30	0.36	0.34	0.41	0.36	0.33	0.42	0.34	0.44	5.6%
Postimmunization	0.01	0.02	0.01	0.01	0.01	0.01	0.02	0.02	0.01	0.02	0.02	8.5%
Toxic	0.03	0.02	0.03	0.01	0.02	0.02	0.03	0.04	0.04	0.04	0.04	3.7%
Other specified	1.17	1.19	1.17	1.30	1.37	1.55	1.40	1.11	1.16	1.26	1.24	0.6%
Viral NOS	0.84	0.89	1.05	1.02	0.93	0.89	0.78	0.83	0.86	0.84	0.81	−0.3%
Unspecified	2.34	2.41	2.78	2.89	2.93	2.95	2.81	1.94	2.22	2.37	2.53	0.8%
**Sex**												
Male	6.14	6.39	7.24	7.53	7.29	7.87	7.14	6.33	6.76	6.86	7.71	2.3%
Female	7.04	7.19	7.90	8.18	8.16	8.67	8.15	6.57	7.00	7.17	7.55	0.7%
**Age, years**												
<1	15.09	14.78	12.88	14.32	12.36	16.84	13.04	11.93	11.20	11.29	15.18	0.1%
1–4	3.77	5.35	5.64	5.08	4.98	7.08	5.43	4.73	4.45	3.60	5.91	4.6%
5–19	3.55	4.13	4.41	4.92	4.77	6.07	4.19	3.87	3.61	3.91	4.63	2.7%
20–44	6.00	5.76	6.49	6.58	6.53	5.91	5.90	5.05	5.50	5.20	5.67	−0.6%
45–64	7.68	7.94	8.92	9.56	9.34	10.05	9.08	7.49	7.99	8.37	8.94	1.5%
≥65	12.11	12.22	14.00	14.19	14.08	14.89	15.97	12.78	14.40	15.07	14.70	2.0%
**Region**												
Northeast	6.94	8.29	6.97	7.27	7.67	8.16	8.00	6.41	7.54	6.30	8.03	1.5%
Midwest	6.83	6.18	7.98	8.00	8.28	8.79	8.13	6.02	6.04	6.65	7.38	0.8%
South	6.62	6.93	8.17	8.64	8.30	8.59	7.78	7.12	7.42	7.52	8.30	2.3%
West	6.05	5.97	6.73	7.13	6.49	7.46	6.78	5.94	6.42	7.18	6.53	0.8%

Abbreviations: HSV  =  Human Herpesvirus; VZV  =  Varicella Zoster Virus; HHV  =  Human Herpesvirus; NOS  =  not otherwise specified.

NA  =  Not available due to no observations recorded in the specified year.

a) Annual growth rate is the yearly compounded growth supposing that the rate for year A is X and year B is Y, Growth Rate  =  (Y/X)^{1/(B-A)}-1^

Known and unknown causes of encephalitis each accounted for approximately half of all cases (53.1% vs. 46.9%, respectively) (**Figure 1B**). The leading identified cause of encephalitis was known viral, encompassing 25.6% of all admissions. The most common specific etiology was HSV-1/HSV-2 encephalitis, accounting for 13.8% of encephalitis hospitalizations and 21.8% of encephalitis inpatient deaths.

**Figure 2:**
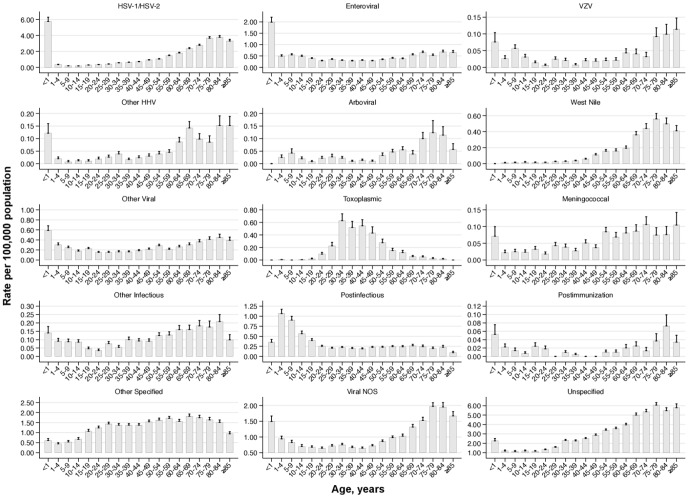
Encephalitis hospitalization rates by five-year age groups stratified by disease category^a^. Abbreviations: HSV  =  Herpes Simplex Virus; VZV  =  Varicella Zoster Virus; HHV  =  Human Herpesvirus; NOS  =  not otherwise specified. a) Population rates are calculated using hospitalization estimates from the Nationwide Inpatient Sample and population-specific estimates from the *United States Census Bureau*. Toxic encephalitis not shown here.

In examining rates by etiology in encephalitis inpatients, there are notable differences between genders and across age groups. Toxoplasmic encephalitis and West Nile Virus were each approximately two-fold more prevalent in males than females (**Table 4**). Other Specified causes, comprised chiefly of immune-mediated causes (see: **Table 1**), were more prevalent in the female (1.69; 95% CI: 1.62–1.76 per 100,000) vs. male population (0.82; 95% CI: 0.78–0.86 per 100,000). Toxoplasmic encephalitis was found primarily in middle-aged populations, the human herpes viruses (HSV-1/HSV-2, VZV, and other HHV) more commonly occurred in those at the extremes of age (i.e., age <1 year and ≥65 years), arboviral and West Nile Virus encephalitis occurred at higher rates in elderly individuals, and enteroviral and postinfectious encephalitis were overrepresented in the pediatric population (**Figure 2**).

**Table 4:** Encephalitis sex, age, and region-specific population rates^a^.

	Hospitalization rates per 100,000 population ± SE														
	HSV-1&2	Entero-viral	VZV	Other HHV	Arboviral	West Nile	Other viral	Toxo-plasmic	Meningo-coccal	Other inf.	Postinf.	Post-immun.	Other spec.	Viral NOS	Unspec
All Cases	1.01±0.03	0.42±0.02	0.03±0.003	0.04±0.003	0.03±0.004	0.11±0.007	0.23±0.01	0.21±0.04	0.05±0.003	0.10±0.005	0.35±0.02	0.01±0.002	1.27±0.05	0.88±0.03	2.56±0.08
Sex															
Male	1.02±0.04	0.49±0.02	0.03±0.004	0.04±0.005	0.04±0.005	0.15±0.010	0.25±0.01	0.28±0.050	0.06±0.005	0.11±0.007	0.34±0.02	0.01±0.003	0.82±0.04	0.92±0.03	2.43±0.08
Female	1.01±0.03	0.35±0.02	0.03±0.003	0.04±0.004	0.03±0.003	0.07±0.006	0.21±0.01	0.15±0.032	0.04±0.004	0.08±0.006	0.35±0.02	0.01±0.003	1.69±0.07	0.84±0.03	2.67±0.09
Age, years															
<1	5.76±0.46	1.99±0.22	0.08±0.028	0.12±0.039	NA	NA	0.59±0.09	NA	0.07±0.029	0.14±0.038	0.35±0.07	0.05±0.023	0.61±0.09	1.49±0.16	2.26±0.22
1–4	0.34±0.05	0.51±0.06	0.03±0.009	0.02±0.008	0.02±0.011	0.01±0.006	0.31±0.04	NA	0.02±0.008	0.09±0.017	1.09±0.10	0.02±0.008	0.45±0.06	0.96±0.08	1.20±0.10
5–19	0.21±0.02	0.47±0.04	0.03±0.005	0.01±0.003	0.02±0.008	0.02±0.004	0.22±0.02	0.01±0.005	0.03±0.005	0.07±0.009	0.62±0.06	0.02±0.004	0.76±0.07	0.73±0.04	1.16±0.07
20–44	0.53±0.02	0.31±0.01	0.02±0.003	0.02±0.003	0.02±0.003	0.03±0.004	0.16±0.01	0.41±0.073	0.04±0.004	0.07±0.006	0.21±0.01	0.01±0.002	1.36±0.06	0.68±0.03	2.00±0.07
45–64	1.24±0.05	0.34±0.02	0.02±0.004	0.05±0.006	0.03±0.005	0.15±0.013	0.24±0.02	0.26±0.056	0.07±0.007	0.12±0.010	0.23±0.02	0.01±0.002	1.61±0.07	0.87±0.03	3.38±0.11
≥65	3.04±0.10	0.60±0.03	0.07±0.011	0.12±0.014	0.08±0.016	0.45±0.033	0.37±0.02	0.03±0.009	0.09±0.011	0.17±0.017	0.22±0.02	0.03±0.007	1.60±0.07	1.64±0.06	5.47±0.19
Year															
2000	0.89±0.06	0.34±0.03	0.05±0.010	0.00±0.000	0.01±0.003	0.00±0.000	0.23±0.03	0.33±0.077	0.05±0.010	0.07±0.014	0.26±0.04	0.01±0.004	1.17±0.09	0.84±0.05	2.34±0.13
2005	1.17±0.07	0.49±0.05	0.03±0.007	0.00±0.000	0.04±0.015	0.25±0.031	0.25±0.03	0.14±0.025	0.04±0.009	0.07±0.012	0.40±0.05	0.01±0.004	1.55±0.13	0.89±0.05	2.95±0.17
2010	1.11±0.07	0.52±0.06	0.03±0.008	0.14±0.019	0.04±0.015	0.14±0.026	0.26±0.03	0.14±0.042	0.06±0.010	0.13±0.016	0.44±0.06	0.02±0.006	1.24±0.10	0.81±0.06	2.53±0.16
Region															
Northeast	1.09±0.09	0.33±0.02	0.04±0.008	0.04±0.007	0.02±0.006	0.04±0.008	0.28±0.03	0.35±0.072	0.05±0.008	0.14±0.015	0.42±0.06	0.01±0.004	1.29±0.13	0.80±0.07	2.49±0.20
Midwest	0.99±0.06	0.45±0.03	0.03±0.006	0.03±0.005	0.05±0.010	0.13±0.015	0.21±0.02	0.06±0.010	0.04±0.006	0.08±0.009	0.35±0.04	0.02±0.004	1.11±0.09	1.12±0.07	2.59±0.16
South	1.09±0.06	0.45±0.03	0.02±0.004	0.05±0.007	0.04±0.007	0.10±0.011	0.24±0.02	0.32±0.105	0.05±0.005	0.10±0.010	0.33±0.04	0.01±0.002	1.29±0.09	0.85±0.05	2.80±0.16
West	0.84±0.05	0.42±0.02	0.02±0.004	0.03±0.006	0.01±0.002	0.15±0.017	0.21±0.02	0.08±0.013	0.05±0.007	0.08±0.010	0.34±0.04	0.01±0.003	1.37±0.11	0.78±0.05	2.19±0.14

Abbreviations: SE  =  standard error; HSV  =  Herpes Simplex Virus; VZV  =  Varicella Zoster Virus; HHV  =  Human Herpesvirus; inf.  =  infectious; immune.  =  immunization; Spec.  =  Specified; NOS  =  not otherwise specified; Unspec.  =  Unspecified.

NA  =  not available due to observations ≤10 admissions suppressed in compliance with Agency for Healthcare Research and Quality guidelines.

a) Toxic encephalitis not shown here (overall hospitalization rate  =  0.03 per 100,000 population).

**Figure 3:**
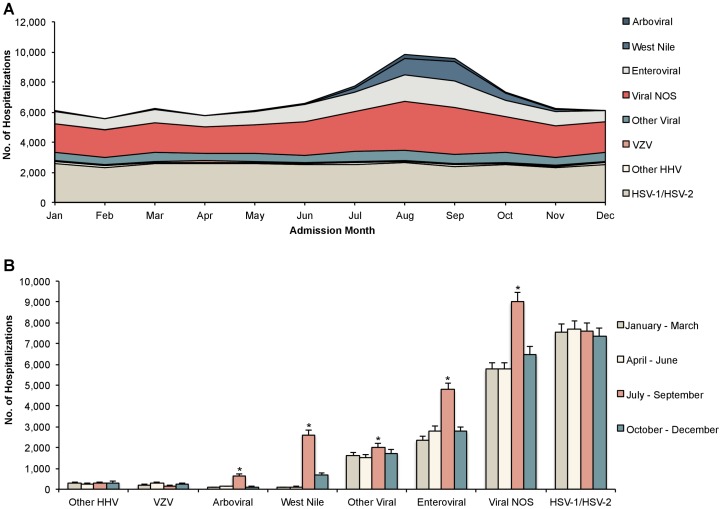
Seasonal variation of hospital admissions for viral encephalitis (A) by month^a^ and (B) by season^b^ (n = 83,225). Abbreviations: NOS  =  not otherwise specified; VZV  =  Varicella Zoster Virus; HHV  =  Human Herpesvirus; HSV  =  Herpes Simplex Virus. a) Includes a stacked representation of all known viral and viral NOS hospital admissions for encephalitis from 2000 to 2010. For example, there was an estimated total of 9,827 viral and viral NOS admissions in the month of August over the entire 11-year period from 2000 to 2010, of which, 1,085 (11%) were due to West Nile Virus infection. Data on admission month was missing for 8.7% of encephalitis admissions. b) Significance: *P<.01 in both base case (2000–2010) and sensitivity analysis (2005–2010) using adjusted Wald test comparing each interval to January-March. Under the base case analysis, July-September for other viral causes and October-December for viral NOS causes were significant (*P*<.05); however, these were insignificant in the sensitivity analysis (*P* = .12 and *P* = .75, respectively).

Substantial seasonal variation was observed in patients presenting with viral encephalitis between 2000 and 2010 (**Figure 3A**). Viral NOS cases were also included to determine if seasonal distribution was similar to arboviruses. Arboviral, West Nile Virus, enteroviral, and viral NOS causes exhibited an increase in admissions from July-September (**Figure 3B**). In addition, seasonal increases in West Nile Virus encephalitis extended into the fall months of October and November. While viral NOS causes also demonstrated an increase in October-December in the 11-year aggregate analysis, this increase becomes insignificant (*P* = 0.75) in a sensitivity analysis examining years for which West Nile Virus was separately reported (i.e., 2005–2010).

Patients admitted to inpatient care stayed an average of 11.2±0.2 days (Median  = 7; IQR  = 9). Length of stay increased over the study period from 10.6 days in 2000 to 12.6 days in 2010 (*P*<0.001). Approximately one in twenty encephalitis admissions resulted in in-hospital mortality (5.6%), with no change in mortality over the study period. During hospitalization, 14.5% of patients required intubation and mechanical ventilation, 9.1% developed sepsis, and 4.5% had aspiration pneumonia, each associated with increased mortality (**Table 5**). Unadjusted mortality was lowest for privately insured patients and highest among Medicare patients (3.7% vs. 8.5%, *P*<0.001). Patients whose primary payer was classified as self-pay, no charge or other experienced a rate of mortality similar to that found among patients covered by Medicaid (5.5% vs. 5.4%, P = 0.784); however, these patients represent a highly heterogeneous group making this result difficult to interpret. Crude mortality by etiology was highest for meningococcal disease (10.3%), other infectious encephalitis (9.3%), HSV-1/HSV-2 (8.9%), and toxoplasmic encephalitis (8.8%).

**Table 5:** Mortality of encephalitis hospitalizations, 2000–2010.

Variable	Mortality^a^								
	No.^b^	Unadjusted rate, per 100 admissions	Univariate, OR (95% CI)		*P* value	Multivariate, OR (95% CI)^c^			*P* value
All Cases	13,464	5.6	-			-			
Gender									
Male	6,893	6.1	1.00	[Reference]		1.00	[Reference]		
Female	6,572	5.2	0.84	(0.78–0.91)	<.001	0.99	(0.88–1.11)		0.850
Age, years									
<1	262	4.4	2.43	(1.78–3.32)	<.001	2.66	(1.72–4.11)		<.001
1–4	248	2.8	1.50	(1.07–2.11)	0.030	1.59	(0.94–2.69)		0.081
5–19	562	1.9	1.00	[Reference]		1.00	[Reference]		
20–44	2,912	4.3	2.37	(1.93–2.92)	<.001	1.84	(1.30–2.60)		0.001
45–64	3,889	5.6	3.11	(2.54–3.80)	<.001	2.41	(1.73–3.34)		<.001
≥65	5,592	9.7	5.64	(4.61–6.90)	<.001	4.91	(3.53–6.82)		<.001
Race									
White	6,804	6.0	1.00	[Reference]		-			
Black	2,238	6.4	1.07	(0.95–1.21)	0.420	-			
Hispanic	1,159	5.1	0.84	(0.72–0.97)	0.020	-			
Asian, Pacific Islander, Native American, or other	854	6.5	1.09	(0.92–1.29)	0.450	-			
Missing	2,410	4.4	-		<.001	-			
Primary payer									
Medicare	6,103	8.5	1.00	[Reference]		-			
Private	3,609	3.7	0.41	(0.37–0.46)	<.001	-			
Medicaid	2,454	5.4	0.62	(0.55–0.70)	<.001	-			
Self pay, no charge, or other	1,266	5.5	0.63	(0.55–0.73)	<.001	-			
Charlson Comorbidity Index^d^									
Mild	3,414	3.0	1.00	[Reference]		1.00	[Reference]		
Moderate	2,769	5.6	1.88	(1.67–2.12)	<.001	1.42	(1.20–1.69)		<.001
Severe	7,282	9.6	3.37	(3.06–3.72)	<.001	2.05	(1.71–2.46)		<.001
Comorbidities									
Cancer^e^	2,371	14.7	3.27	(2.94–3.66)	<.001	2.26	(1.88–2.71)		<.001
HIV/AIDS	1,830	9.9	1.94	(1.73–2.19)	<.001	1.70	(1.30–2.22)		<.001
Complications									
Respiratory Intubation and Mechanical Ventilation	7,951	31.8	10.70	(9.87–11.7)	<.001	-			
Sepsis	4,437	17.7	5.90	(5.37–6.49)	<.001	-			
Aspiration Pneumonia	2,041	16.8	4.48	(4.01–5.02)	<.001	-			
Hospital Region									
Northeast	2,892	6.5	1.00	[Reference]		1.00	[Reference]		
Midwest	2,453	4.6	0.70	(0.60–0.81)	<.001	0.77	(0.64–0.94)		0.010
South	5,059	5.5	0.84	(0.74–0.95)	0.005	0.92	(0.79–1.09)		0.340
West	3,060	6.2	0.95	(0.82–1.09)	0.550	1.06	(0.89–1.27)		0.526
Hospital Location/Teaching Status									
Rural	908	4.3	1.00	[Reference]		1.00	[Reference]		
Urban, Nonteaching	4,474	5.7	1.35	(1.15–1.59)	<.001	1.47	(1.16–1.86)		0.001
Urban, Teaching	8,024	5.8	1.39	(1.18–1.62)	<.001	1.88	(1.50–2.37)		<.001
Encephalitis Cause									
HSV-1/HSV-2	2,937	8.9	1.00	[Reference]		1.00	[Reference]		
Enteroviral	520	3.8	0.40	(0.33–0.49)	<.001	0.68	(0.55–0.83)		<.001
VZV	44	4.6	0.49	(0.24–0.99)	0.047	0.53	(0.26–1.09)		0.085
Other HHV	102	7.8	0.85	(0.53–1.37)	0.516	0.71	(0.44–1.15)		0.161
Arboviral	49	4.8	0.55	(0.29–1.04)	0.068	0.67	(0.36–1.26)		0.211
West Nile	271	7.7	0.85	(0.65–1.13)	0.265	0.86	(0.64–1.15)		0.306
Other viral	458	6.0	0.65	(0.51–0.83)	0.001	-			
Toxoplasmic	609	8.8	0.98	(0.78–1.23)	0.872	-			
Meningococcal	160	10.3	1.17	(0.81–1.69)	0.403	1.41	(0.95–2.08)		0.084
Other infectious	296	9.3	1.04	(0.77–1.40)	0.797	-			
Postinfectious	303	2.7	0.28	(0.22–0.36)	<.001	0.56	(0.42–0.75)		<.001
Postimmunization	NA	NA	0.12	(0.02–0.90)	0.039	0.15	(0.02–1.01)		0.051
Toxic	45	4.8	0.51	(0.26–1.00)	0.049	0.49	(0.24–0.99)		0.046
Other Specified	2,157	5.2	0.56	(0.50–0.64)	<.001	0.66	(0.57–0.75)		<.001
Viral NOS	1,094	3.8	0.40	(0.34–0.47)	<.001	-			
Unspecified	4,421	5.3	0.57	(0.51–0.64)	<.001	-			

Abbreviations: SE  =  standard error; OR  =  Odds Ratio; CI  =  Confidence Interval; HIV/AIDS  =  Human Immunodeficiency Virus/Acquired Immunodeficiency Syndrome; HSV  =  Herpes Simplex Virus; VZV  =  Varicella Zoster Virus; HHV  =  Human Herpesvirus; NOS  =  not otherwise specified.

NA  =  not available due to observations ≤10 admissions suppressed in compliance with Agency for Healthcare Research and Quality guidelines.

a) 392 admissions (n = 2,364 weighted) with missing death, sex, or hospital location/teaching status were excluded.

b) Total deaths were weighted to represent the entire US population using discharge weights provided by the Nationwide Inpatient Sample.

c) The logistic regression model excludes the following disease categories: Unspecified, Other viral, Other infectious, Viral NOS, and Toxoplasmosis (n = 26,232; 128,679 weighted); Final unweighted n = 21,972, weighted n = 107,985. Model constant = 0.012 ± 0.003; Model calibration: Hosmer and Lemeshow goodness-of-fit test: χ^2^  =  1.803, df  =  9, *P* = 0.063.

d) The Charlson Comorbidity Index is a weighted index based on the presence of 17 comorbid conditions. Each comorbid disease is assigned a weight from 1 to 6; the index is the sum of the weights for each comorbid condition and can range from 0 to 33. For the purposes of this study, mild was score  =  0, moderate score  =  1, and severe score ≥ 2.

e) Cancer deaths include both metastatic (30%) and nonmetastatic (70%); crude mortality rates are 15.1% and 14.5%, respectively.

In multivariable regression, patients with encephalitis and comorbid HIV/AIDS had a more than 50% increased odds of in-hospital mortality compared to those without HIV/AIDS (odds ratio [OR]  = 1.70; 95% CI: 1.30–2.22) (**Table 5**). Although toxoplasma infection was excluded from the model due to high collinearity with HIV/AIDS, there was no difference in unadjusted mortality in toxoplasma patients with and without HIV (9.4% vs. 14.1%; *P* = 0.32). Patients with comorbid cancer had a two-fold increase in the odds for adjusted in-hospital mortality compared to those without (OR = 2.26; 95% CI: 1.88–2.71).

In the same multivariable model, compared with those 5-19 years old, individuals ≥65 years of age demonstrated the greatest risk of mortality (OR = 4.91; 95% CI: 3.53–6.82) while newborns and infants <1 year old and individuals aged 45–64 years each demonstrated a nearly three-fold higher odds of mortality (OR = 2.66; 95% CI: 1.72–4.11 and OR = 2.41; 95% CI: 1.73–3.34, respectively).

Patients with postinfectious, toxic, and Other Specified causes of encephalitis demonstrated lower odds of inpatient mortality compared with patients suffering from herpes simplex encephalitis (**Table 5**). Mortality among patients with other known viral encephalitides (e.g., West Nile Virus, VZV) did not differ substantially from herpes simplex encephalitis. Although crude mortality for meningococcal encephalitis was high, adjusted mortality did not differ significantly from herpes simplex encephalitides (OR = 1.41; 95% CI: 0.95–2.07; *P* = 0.09).

## Discussion

Our findings demonstrate that the burden of disease associated with encephalitis in the United States, including mortality and associated factors, remains substantial, although demographics have changed from prior decades. The overall rate of hospitalization, 7.3 per 100,000 population, was not different from that reported in the U.S. over ten years earlier [4], nor did the rate differ from that reported by Vora et al. (2014) [6]; however, it was higher than reported rates from hospital discharge data in France, Italy, Canada, and Australia [22]–[25]. Modest reductions were observed in the mean length of stay and in-hospital mortality compared to findings from the previous decade [4]. However, length of stay for those admitted with encephalitis trended upward across the decade, in contrast to the general trends in the U.S. with respect to other diseases [18], [26]–[28]. Viral causes continue to account for the majority of cases in which an etiology is identified, although a substantial proportion of cases are now associated with Other Specified (predominantly autoimmune) causes. As has been the case in numerous studies spanning the last several decades, approximately 50% of cases had a specific etiology assigned [1], [2], [4], [23]–[25], [29].

The reported demographics of hospitalized encephalitis patients in the U.S. are changing. The encephalitis-associated hospitalization rate for females was higher than for males (7.6 female vs. 7.0 male per 100,000 population), while in the previous decade rates were found to be substantially higher in males (6.4 female vs. 8.2 male per 100,000 population) [4]. Several factors likely contribute to this observation. First, hospitalization rates of toxoplasma encephalitis, a disease predominantly of males, has declined with the widespread use of effective antiretroviral treatment for HIV [30]. In addition, hospitalization rates of those with Other Specified causes, in which rates are twice as high in females compared with males (1.7 vs. 0.83 per 100,000 population), have increased. This etiologic group is comprised predominantly of autoimmune encephalitides such as anti-NMDA-receptor encephalitis, which is known to be more common in women [3], [31]. Of note, a trend for increased encephalitis-associated hospitalizations in women was also recently reported in Canada. This increase was attributed largely to an increased incidence of VZV encephalitis among women [25], which was not observed in our study. Thus, our findings of a change in gender-specific hospitalization rates associated with encephalitis may reflect, in part, increased awareness, diagnosis and coding of autoimmune encephalitides.

The age-specific rates of encephalitis-associated hospitalizations in our study were similar to previous reports, with the exception of meningococcal encephalitis. As in previous studies, we found the highest rates of hospitalization for all-cause and herpes encephalitis at extremes of age [4], [23], [25]. Rates of West Nile Virus encephalitis-associated hospitalizations were lowest in those 19 years of age and under, higher in the 45–64 age group, and highest in those 65 and over, paralleling West Nile Virus national surveillance data collected through the passive surveillance system ArboNET by the *Centers for Disease Control and Prevention* from 1999–2008 [7]. Unexpectedly, rates of meningococcal encephalitis-associated hospitalizations were as high in those 65 years of age and older (0.09 per 100,000) as those less than 1 year of age (0.07 per 100,000), despite an almost 10-fold lower incidence of overall meningococcal infection in the elderly during a similar study period [32]. Several factors may contribute to the discrepancy between rates of infection and rates of encephalitis-associated hospitalization. Possibilities include increased risk of central nervous system (CNS) disease in elderly individuals infected with meningococcus, as well as increased severity of illness in the elderly resulting in a higher percentage who are hospitalized, supported by the higher case fatality rate among older individuals [32], [33]. Nevertheless, these data suggest that the elderly, in addition to the very young and teenagers, are at risk for severe, invasive CNS meningococcal disease. Improving the timely recognition of this condition in the elderly may accelerate implementation of measures crucial for effective treatment such as earlier antibiotic and supportive therapy, while also prompting initiation of prophylaxis for contacts [34].

The spatial and temporal patterns of encephalitis-associated hospitalizations suggest that arboviral encephalitis may be underdiagnosed in the U.S. Of the various etiological groups, only viral NOS and arboviral encephalitis demonstrated differential region-specific rates of hospitalization in the following descending order: Midwest > South > Northeast > West (**Table 1**). Moreover, examination of the seasonality of hospitalization rates demonstrates a marked similarity between viral NOS and arboviruses (**Figure 2**). Rates for both increase slightly in March, with a much larger peak between July and October; this is in sharp contrast to HSV1/2 and several other viruses, whose hospitalization rates do not vary by season. Thus, both regional and seasonality data suggest that a substantial proportion of viral NOS cases may in fact be due to unrecognized arboviruses. These results parallel a recent study in Canada in which seasonal clusters of encephalitis were identified, suggesting that unidentified arboviral agents may underlie undetermined etiologies of encephalitis [25]. Taken together, these findings highlight the need for increased surveillance and improved diagnostic modalities to identify arboviral encephalitis. Until more precise diagnostic tools become available, however, clinicians should take care not to allow external factors to bias their reporting; for example, a diagnosis of viral encephalitis NOS should not be presumed in patients presenting in the fall with unknown cause.

Our findings that the extremes of age and multiple comorbidities including HIV/AIDS, cancer, and other medical diseases are associated with increased mortality in encephalitis are in agreement with other studies [2], [8], [35]–[38]. Complications including intubation, acute respiratory failure, aspiration pneumonia, and sepsis were strong predictors of mortality in our study, consistent with a study in France of patients with infectious encephalitis [2]. Similarly, in patients with encephalitis of all causes admitted to a critical care unit, intubation, cerebral edema, status epilepticus, and thrombocytopenia predicted mortality [39]. Moreover, we found that several etiologies, including post-infectious and Other Specified groups, were associated with decreased mortality when compared to herpes simplex encephalitis, while meningococcal encephalitis demonstrated the greatest unadjusted mortality. Other infectious agents have also been associated with increased in-hospital mortality compared to herpes encephalitis [2], although differences in etiologic spectrum preclude a direct comparison with our findings.

This study has several important limitations. All studies which rely on administrative data not originally collected for research purposes are subject to challenges associated with accuracy, granularity and completeness of diagnosis and procedure coding [40]. However, a recent study comparing encephalitis diagnoses in administrative data to prospectively collected data in a cohort of patients with encephalitis demonstrated that, with the exception of rare infectious agents not specifically associated with an available diagnosis code, reasonable concordance between administrative and prospective data existed, supporting the use of administrative data as a limited-cost surveillance tool [24]. In our study, no physiologic information was available beyond that which can be gleaned from ICD-9 codes, and potentially important unreported factors related to prognosis, treatment choice and outcome could not be assessed. More specific diagnostic coding (e.g., autoimmune encephalitis) and linkages with prescription drug databases may improve the quality and broaden the scope of analyses possible with administrative datasets such as the NIS. Also, because the NIS is built upon a weighted sample encompassing approximately 20% of all acute-care hospital discharges annually, our population-level estimates may be imprecise because of unmeasured differences in case-mix between NIS participating hospitals and non-participating facilities. This may be especially true for the more rarely reported conditions. In addition, the NIS dataset does not permit the study of individual patient or hospitalization death records, which limits our analysis to mortality of encephalitis-associated hospitalizations, rather than known encephalitis-associated deaths. Individual patient identifiers are not available in the database; therefore, it may be possible for a single patient to be counted twice within the sample, which could lead to overestimation of incidence.

The strength of this report lies in the very large number of patients available for study, which has permitted a comprehensive examination of acute in-patient hospitalizations for encephalitis at the national level across a decade. Our results, which build upon those of previous studies [4], [6], provide further insight into temporal changes in the etiology of encephalitis. Also, our findings corroborate earlier reports that encephalitis remains an important public health issue and that the burden associated with encephalitis, including patient mortality, has changed little across the past two decades [4], [6]. Despite advances in technology and pharmacology, encephalitis remains challenging to diagnose and difficult to treat. The fact that no etiologic agent or process is identified in 50% of encephalitis patients is troubling and clearly points to the need for further research. In addition, developing more specific and granular diagnostic coding for inclusion in administrative data would improve our ability to understand the epidemiology of encephalitis at the population level. While outcomes beyond hospital discharge could not be examined using the cross-sectional data available for this study, it remains important to develop a more complete understanding of long-term outcomes among encephalitis patients. Our findings will serve as a 21^st^ Century benchmark for future interpretation of the epidemiology of encephalitis and encephalitis-related in-hospital mortality.
